# Rapid Decision-Making with Side-Specific Perceptual Discrimination in Ants

**DOI:** 10.1371/journal.pone.0012377

**Published:** 2010-08-24

**Authors:** Nathalie Stroeymeyt, Fernando J. Guerrieri, Jelle S. van Zweden, Patrizia d'Ettorre

**Affiliations:** Department of Biology, Centre for Social Evolution, University of Copenhagen, Copenhagen, Denmark; Vrije Universiteit, Netherlands

## Abstract

**Background:**

Timely decision making is crucial for survival and reproduction. Organisms often face a speed-accuracy trade-off, as fully informed, accurate decisions require time-consuming gathering and treatment of information. Optimal strategies for decision-making should therefore vary depending on the context. In mammals, there is mounting evidence that multiple systems of perceptual discrimination based on different neural circuits emphasize either fast responses or accurate treatment of stimuli depending on the context.

**Methodology/Principal Findings:**

We used the ant *Camponotus aethiops* to test the prediction that fast information processing achieved through direct neural pathways should be favored in situations where quick reactions are adaptive. Social insects discriminate readily between harmless group-members and dangerous strangers using easily accessible cuticular hydrocarbons as nestmate recognition cues. We show that *i)* tethered ants display rapid aggressive reactions upon presentation of non-nestmate odor (120 to 160 ms); *ii)* ants' aggressiveness towards non-nestmates can be specifically reduced by exposure to non-nestmate odor only, showing that social interactions are not required to alter responses towards non-nestmates; *iii)* decision-making by ants does not require information transfer between brain hemispheres, but relies on side-specific decision rules.

**Conclusions/Significance:**

Our results strongly suggest that first-order olfactory processing centers (up to the antennal lobes) are likely to play a key role in ant nestmate recognition. We hypothesize that the coarse level of discrimination achieved in the antennal lobes early in odor processing provides enough information to determine appropriate behavioral responses towards non-nestmates. This asks for a reappraisal of the mechanisms underlying social recognition in insects.

## Introduction

Throughout their lives, animals have to assess and integrate information from their environment to modulate their behavior and make informed, optimal decisions. Efficient information processing is therefore crucial for success in a permanently fluctuating environment. Many vertebrate and invertebrate groups have evolved specialized nervous structures dedicated to the processing of signals and cues detected by their sensory organs. Information processing and decision-making can be subject to a typical speed-accuracy trade-off [1]–[6]. Optimal processing strategies and their associated neural pathways may therefore vary depending on whether fast reactions are required (e.g. in life-threatening situations), or slow but accurate treatment of information is needed [3], [7]–[9]. This is the case for both visual and auditory systems in mammals, which are equipped with dual decision-making systems implemented by distinct neural pathways. In both cases, a direct pathway (thalamic pathway for visual stimuli related to fear [10]–[12]; subcortical pathway for auditory fear conditioning [7]) involving coarse, but rapid processing of information, can bypass a slower, highly integrated cortical pathway in order to provide quicker, but less well informed decisions. This is likely to have a high impact on fitness when fast reactions are necessary. We could therefore expect dual processing systems to occur across taxa and across sensory modalities; however, such studies in non-mammals are sparse. One such example was described in bee visual search: the use of a slow chromatic channel or of an alternative faster achromatic channel indeed allows foraging bees to compromise between detection speed and accuracy depending on flower size [13]–[14].

Among the various communication modalities, the chemical channel is one of the most extensively used, therefore chemosensory (i.e. olfactory and gustatory) pathways have been intensively studied in animals [9], [15]. The architecture of olfactory pathways in insects and vertebrates shows many similarities. Olfactory receptor neurons grouped in chemosensory organs converge into glomeruli in first-order integration centers in the central nervous system (CNS): the olfactory bulbs (vertebrates) or the antennal lobes (insects). Information is then usually transferred towards higher-order integration CNS centers, the olfactory cortex (vertebrates), or the mushroom bodies and lateral horns (insects), which coordinate most behavioral and physiological responses [9], [16]. Olfaction is usually characterized by higher response time than vision and audition, both because stimulus propagation is slower and because it involves complex spatial and temporal integration of information [8], [17]–[21]. However, recent studies have shown that simple olfactory binary discrimination tasks can take as little as 200 ms in mice [17], 300 ms in rats [22], and from 400 ms [23] to 690 ms [24] in tethered and free flying honeybees, respectively. It has been proposed that such simple perceptual discrimination tasks are achieved in first-order olfactory processing centers, whereas higher-order processing centers would be responsible for more complex and time-consuming information integration [6]. We therefore expect first-order olfactory integration centers to play a major role in determining behavioral responses in risky situations.

Ants live in societies of hundreds of individuals sharing valuable resources essential for colony reproduction. These must be defended by efficiently distinguishing strangers (“non-nestmates”) from colony members (“nestmates”). Ants do so using colony-specific multi-component chemical cues, the cuticular hydrocarbon (CHC) profiles, detected by their antennae. Ant bodies are indeed covered with a layer of chemicals including varied long-chain hydrocarbons, many of which were shown to play a major role in nestmate recognition [25]–[28]. CHC profiles are complex and dynamic, and vary qualitatively among species and quantitatively within species: colonies of the same species share the same CHCs but differ in their relative proportions [25]–[29]. Nestmate recognition therefore requires fine discrimination of complex CHC mixtures differing in the relative amounts of many compounds. Comparison of multi-component mixtures and identification of individual components from such mixtures have been reported to be complex olfactory tasks requiring longer response times than simpler binary discrimination [6]. However, upon intrusion by competitors or parasites, fast reactions are essential to defend and protect the colony because once an intruder has succeeded to enter the nest it is unlikely to be detected at all [30]. We evaluated the speed of aggressive responses upon presentation of non-nestmate odors in the ant *Camponotus aethiops*, and explored whether essential cue integration steps take place at an early stage in the olfactory system by investigating the side-specificity of responses to non-nestmate odors. Ants are usually very aggressive towards non-nestmates, but they may become more tolerant when allowed prolonged contact with them [31] or with their colony odor [32]. This could be the result of basic processes such as habituation (non-associative, elemental learning resulting in a decrease in responsiveness to a prolonged or repeatedly experienced stimulus [33]) or sensory adaptation, at the receptor level [34]. We designed two procedures of exposure to colony-specific CHCs enabling us to manipulate nestmate recognition, then investigated whether recognition cue processing is side-specific in *C. aethiops* ants.

## Results

### Response times for nestmate recognition

Restrained *C. aethiops* ant workers did not respond to nestmate odor (n = 10), but opened their mandibles (first aggressive display) within 120 to 160 ms of presentation of non-nestmate odor. Six out of 10 ants opened the mandibles within 120 milliseconds and four ants opened the mandibles within 160 milliseconds. This time range indicates an extremely rapid reaction, faster than that recorded for binary discriminatory tasks in honeybees (around 400 ms in restrained bees [23] and 690 ms in free flying bees [24]) and even in mammals (200 ms in mice [17], 300 ms in rats [22]).

### Manipulation of nestmate recognition by exposure to CHCs

**Figure 1:**
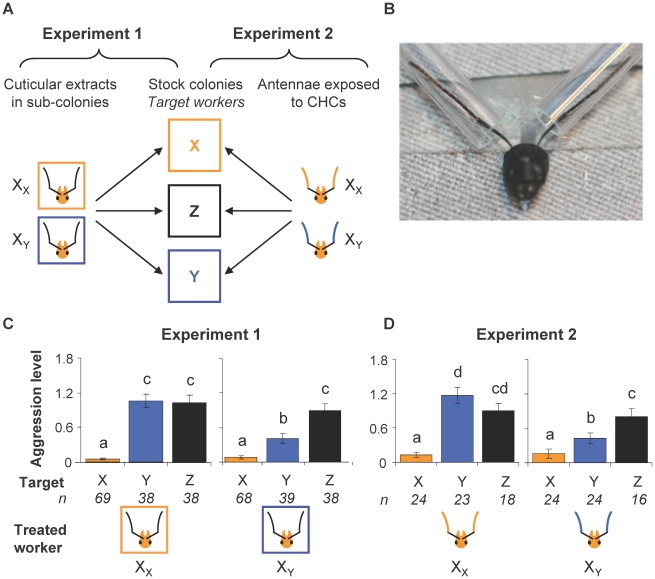
Effect of exposure to alien colony odor on nestmate recognition. (**A**) Experimental design. Workers from colony X were exposed to the odor of either nestmates (X_X_) or non-nestmates from colony Y (X_Y_), either inside sub-colonies during 24 hours (experiment 1) or directly on their antennae during 18 hours (experiment 2). Aggression tests between treated workers and anaesthetized target workers from colonies X, Y or unrelated alien Z were performed immediately after exposure as indicated by the arrows. (**B**) Restrained worker in the antennal exposure device. The picture shows CHC-coated glass capillaries positioned around the worker's antennae. (**C, D**) Aggression level of treated workers towards targets from colonies X (yellow bars), Y (blue bars) and Z (black bars) in experiments 1 (C) and 2 (D). Columns and error bars indicate mean and standard error of aggression indices respectively. Different letters indicate significant differences between categories (mixed-effects model with least square means post-hoc comparisons, *P*<0.05). X_Y_ workers were significantly less aggressive towards non-nestmates from colony Y than X_X_ workers (X_Y_–Y *vs.* X_X_–Y: *P*<0.0001 in both experiments). However, treatments did not influence aggressiveness towards nestmates (X_X_–X *vs.* X_Y_–X, experiment 1: *P* = 0.808; experiment 2: *P* = 0.837) or non-nestmates from colony Z (X_X_–Z *vs.* X_Y_–Z, experiment 1: *P* = 0. 322; experiment 2: *P* = 0. 416).

We exposed workers to colony-specific odors by introducing CHC-coated microscope slides into sub-colonies for 24 hours (experiment 1) or by positioning CHC-coated glass capillaries over the antennae of individual workers for 18 hours (experiment 2; Figure 1A, B). These two procedures gave similar results (Figure 1C, D): workers exposed to CHCs of an alien colony became significantly less aggressive towards freshly killed non-nestmates from that colony, as compared to workers that were exposed to nestmate CHCs (GLMM, least square means comparisons: p<0.0001). Test workers had therefore familiarized themselves with non-nestmate odor upon prolonged exposure to that stimulus. This familiarization process was specific, as workers exposed to non-nestmate CHCs from a given colony did not lower their aggressiveness towards non-nestmates from a different, unfamiliar colony.

### Side-specificity of nestmate recognition cues processing

**Figure 2:**
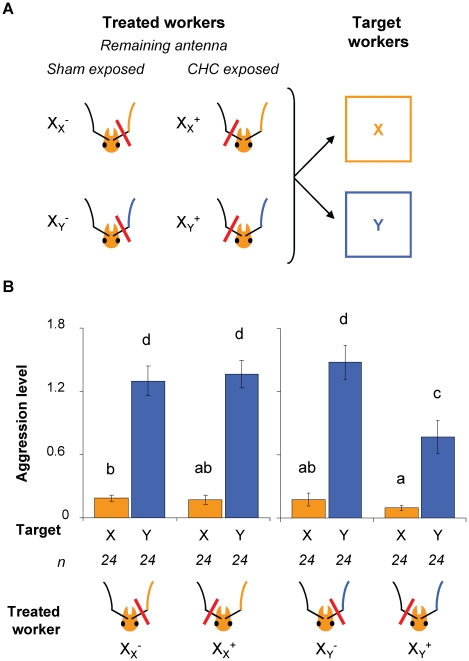
Effect of unilateral antennal exposure to alien colony odor. (**A**) Experimental design. Antennae of workers from colony X were inserted into two capillaries (see also Figure 1B), one of which was treated with solvent (sham exposed, −) while the other was coated with CHCs (CHC-exposed, +) from either nestmate workers (control X_X_) or non-nestmates from colony Y (test X_Y_). After 18-hour exposure, one antenna was selectively excised. Aggression tests between treated workers and anaesthetized target workers from colonies X or Y were performed immediately after excision as indicated by the arrows. (**B**) Aggression level of treated workers towards targets from colonies X (yellow bars) and Y (blue bars). Columns and error bars indicate mean and standard error of aggression indices respectively. Different letters indicate significant differences between categories (mixed-effects model with least square means post-hoc comparisons, *P*<0.05). When their remaining antenna had been exposed to non-nestmate CHCs (X_Y_^+^), workers were significantly less aggressive towards non-nestmates from colony Y than when their remaining antenna had been sham exposed (X_Y_^+^–Y *vs.* X_Y_^−^–Y, *P*<0.0001). X_Y_^+^ workers were also less aggressive towards Y-individuals than control workers, which had been exposed to nestmate odor (X_Y_^+^–Y *vs.* X_X_^+^–Y, *P*<0.0001; X_Y_^+^–Y *vs.* X_X_^−^–Y, *P*<0.0001). The aggressiveness of control workers (X_X_) towards Y-individuals was always high and did not depend on which antenna was excised (X_X_^+^–Y *vs.* X_X_^−^–Y, *P* = 0.821).

We then tested whether the effect of odor familiarization was side-specific or transferred to the other brain hemisphere (experiment 3). Bilateral transfer would be strong evidence for processing in high-order brain centers such as the mushroom bodies [35]–[37], which are highly interconnected between both brain hemispheres [38]. Lack of transfer, on the other hand, would indicate an important contribution from side-specific mechanisms at a lower level, e.g. in the antennae or antennal lobes, which have very few bilateral connections [39]. We exposed workers to colony-specific CHCs on one antenna only (the other antenna being sham treated; Figure 1B), and then selectively ablated one antenna, so that the remaining antenna was either the CHC-exposed (X_X_^+^ and X_Y_^+^) or the sham, solvent-exposed (X_X_^−^ and X_Y_^−^; Figure 2A). When workers from a colony X were unilaterally exposed to the odor of non-nestmates from a colony Y (test workers; X_Y_^+^ and X_Y_^−^), their aggressiveness towards these non-nestmates depended strongly on which antenna had been excised (Figure 2B). Test workers whose remaining antenna was the CHC-exposed (X_Y_^+^) were significantly less aggressive towards Y non-nestmates than all other treated workers (GLMM, least square means comparisons: X_Y_^+^ vs. X_Y_^−^, X_Y_^+^ vs. X_X_^+^ and X_Y_^+^ vs. X_X_^−^, p<0.0001 in all three comparisons). By contrast, test workers whose remaining antenna was sham-exposed (X_Y_^−^) showed similar high level of aggressiveness towards non-nestmates as control workers exposed to nestmate odor (GLMM, least square means comparisons: X_Y_^−^ vs. X_X_^+^, p = 0.774; X_Y_^−^ vs. X_X_^−^, p = 0.608). Unilateral exposure to non-nestmate CHCs therefore induced a behavioral effect similar to bilateral exposure, i.e. a decrease in aggressiveness towards non-nestmates with a familiar odor, but the effect remained restricted to the exposed side and was not transferred to the other brain hemisphere. The perception of non-nestmate odor therefore depended on which antenna was used to detect that odor. On the other hand, the aggressiveness of control workers towards non-nestmates was always high and did not depend on which antenna was excised (GLMM, least square means comparisons: X_X_^+^ vs. X_X_^−^, p = 0.821), which indicates that the prolonged contact with the CHC-coated capillary did not interfere with the detection ability of exposed antennae.

## Discussion

We showed that nestmate recognition in *Camponotus aethiops* ants is characterized by rapid behavioral response time (<160 ms) in spite of the apparent complexity of the olfactory discrimination task involved, i.e. the detection of differences in relative amounts of several compounds in a multi-component chemical signal [28]. This response time was faster than those measured in honeybees and mammals for simpler binary discrimination tasks [17], [21]–[24]. This may seem surprising, as discrimination of complex mixtures usually requires longer information processing [6]. It may be argued that ants may not exploit the totality of their CHC profile, but only use a small subset of compounds for nestmate recognition, which would greatly reduce the complexity of the task. Indeed, several studies have shown that certain classes of hydrocarbons (e.g. linear alkanes in ants [29], wasps [40] and bees [41]–[42]) may not play a role in nestmate recognition. However, other classes of hydrocarbons such as alkenes and branched alkanes have been shown to play a major role in nestmate recognition [29], [40]–[42]. *C. aethiops* CHC-profiles typically have more than 15 different dimethylated alkane compounds clearly distinguishable by gas chromatography coupled with mass spectrometry [43]. Consequently, even if ants only exploit a single class of CHCs, nestmate recognition should be a much more complex task than discrimination of binary mixtures, and the reaction times observed in our study remain surprisingly short. This paradox could be explained if the mechanisms involved in olfactory discrimination differ between nestmate recognition and the binary discrimination tasks mentioned above, emphasizing e.g. either speed or accuracy of responses. The studies on honeybees and mammals were indeed performed in an appetitive context, i.e. subjects were given a food reward if they completed the discrimination task correctly. In that case, task efficiency depends mainly on the accuracy of the answers, and there is little to gain from an increase in speed. Nestmate recognition, on the contrary, requires fast reactions to ensure maximal efficiency in colony defense. It therefore appears that different strategies for information processing may have evolved in the olfactory system of insects to optimize responses in different contexts, just as described for visual and auditory systems in mammals [1], [7], [10]–[12].

We further showed that aggression of *C. aethiops* ants towards non-nestmates can be reduced by prolonged exposure to the colony odor of these specific non-nestmates (familiarization-like process) without requiring social interactions with non-nestmates. In addition, when one antenna is exposed to non-nestmate odor and the other antenna is sham-treated, aggression towards non-nestmate individuals is only reduced when these are detected by the exposed antenna, but not by the sham-treated antenna. This shows that ants do not compare the detected odor to a unique, central representation of colony odor, but rather obey to side-specific decision rules, which can be altered independently in each side. Nestmate recognition does therefore not depend on integrated bilateral transfer of information through the brain, either during familiarization to colony odor or for decision making.

Our results contrast with those obtained after unilateral olfactory conditioning in the honeybee: after conditioning the proboscis extension reflex (PER, a classical paradigm for associative learning) on one antenna only, the authors observed that the learned information was transferred between sides, suggesting the involvement of integration and bilateral transfer in high-order integration centers such as mushroom bodies [35]–[36]. This transfer occurred within 3 hours after unilateral PER conditioning [35]; in our case no bilateral transfer occurred although we allowed ample time for it (18 hours). This is further evidence that the mechanisms and neural substrates involved in information processing and plasticity in nestmate recognition differ from those involved in olfactory discrimination and learning in an appetitive context.

The side-specificity in responses to non-nestmates observed in experiment 3 demonstrates that processing of nestmate recognition cues and nestmate recognition plasticity are side-specific processes. This provides interesting insights on the possible neural substrates involved in nestmate recognition. Such strong side-specificity in responses would indeed be unexpected for a process mainly controlled in highly interconnected high-order brain centers [38]. Conversely, side-specific responses are more likely to occur if they are mainly determined at an early stage in the olfactory system, up to the first synaptic relay in the antennal lobes, as these structures are poorly interconnected [39]. We therefore hypothesize that behavioral responses in the context of nestmate recognition are determined as early as at the level of the antennal lobes, then relayed to the motor centers via higher-level brain centers without requiring further processing to refine discrimination. There could also be a direct connection between antennal lobes and motor centers such as the suboesophageal ganglion, as observed in moths [44]–[45]. Such direct connection would contribute to fast information processing and should be investigated in ants and other social Hymenoptera.

More specifically, we suggest that two non-exclusive neural structures could play a major role in determining behavioral responses in the context of nestmate recognition. Firstly, a prolonged exposure to non-nestmate CHCs could induce sensory adaptation of the antennal receptors, resulting in a decrease in the perception of non-nestmate odor. This hypothesis is in agreement with previous findings in *Camponotus japonicus*, where CHC-sensitive chemosensillae were shown to respond less to nestmate than to non-nestmate CHCs and display decreasing responsiveness upon prolonged exposure to non-nestmate CHCS [46]. Secondly, our observed behavioral response could be mediated by the CNS at the level of the antennal lobes, e.g. through elemental olfactory learning processes such as habituation. This is consistent with findings in *Drosophila* showing that prolonged exposure to an odorant induces structural changes in the antennal lobes correlated with a decrease in responsiveness to that odorant [47]. In honeybees, first divergences between the representations of different odors in the antennal lobes can be observed within tens of milliseconds. This is due to a few projection neurons with low response latencies, which provide an early but incomplete representation of an odor's identity [48]. We suggest that the coarse discrimination level achieved at this early stage could provide enough information to induce rapid behavioral responses when speed prevails over accuracy – which would account for the rapid responses to non-nestmate odors (<160 ms) observed in our study with tethered *C. aethiops* workers.

Further investigations will be required to establish the precise role of the antennal lobes in nestmate recognition cues processing and test the above scenario suggested by our experimental results. The use of selective lesions of parts of the brain (e.g. unilateral lesion of antennal lobes, mushroom bodies or lateral horns) or selective inhibitors may provide interesting insights on which parts of the brain are involved in the familiarization process described in our experiments. Additionally, electrophysiological recordings and neuroimaging on tethered ants presented with nestmate or non-nestmate odors may be very useful to detect the spatial and temporal pattern of activation of different parts of the brain.

Altogether, our results suggest a novel scenario for processing of nestmate recognition cues in social insects. In natural conditions, ants are permanently exposed to their own colony odor. This prolonged exposure induces familiarization to nestmate odor, either at the level of the antennae [46] or the antennal lobes. As a result, individuals do not usually respond to nestmate odor, but will display very fast responses to any novel, unfamiliar odor. This provides a parsimonious explanation to the observations that (*i*) unfamiliar CHC patterns (including both quantitative and qualitative differences from the colony odor) trigger aggression, and (*ii*) this aggressive response fades after prolonged exposure [31]–[32]. This model is in agreement with a recent study on nestmate recognition in a related *Camponotus* species, which showed that workers specifically reject individuals bearing odor cues that are novel to their own colony cuticular hydrocarbon profile, but do not reject those lacking one compound [29]. Moreover, our model can help explaining the mechanisms underlying the chemical integration of social parasites into host colonies [49] and the pacific co-existence of different ant species with distinct cuticular profiles in arboreal ant gardens [50]. In both cases, it is likely that workers familiarize themselves with the odor of their social parasites or of their parabiotic partners upon repeated contact with them, therefore showing lowered levels of aggressiveness, even if both odors do not exactly match [51]. Social parasites may then passively acquire (camouflage) and/or actively synthesize (mimicry) the recognition cues of the host colony, therefore expressing a new odor more similar to that of their host colony [49].

We thus suggest a reappraisal of the common interpretation of the mechanisms underlying the “template-label matching model” and the “bar-code” hypothesis [25]. It has been assumed for a long time that ants and other social insects compare a set of cues, the label (CHC-profile of a given individual analogous to a bar-code), with a learned template (inner representation of the colony-specific CHC-profile stored in the long-term memory) [28], [32], [52]–[53]. This implies that for an appropriate behavioral response to occur, the intruder's chemical profile must be matched “point by point” against the learned colony odor template. Its antennae would act as a bar-code reader device which passes the information on to a central processing unit where the matching occurs [25]. Under this still widespread interpretation (see e.g. [54]), we would expect the aggressive response to follow a unique, non side-specific decision rule, and workers to react to all mismatches in the hydrocarbon profile, including the absence of a compound – which was not observed when tested [29].

Olfactory discrimination has been shown to be a patterned, time-dependent process, whereby differences in odor representation in the brain centers increase over time [6], [48]. In the context of nestmate recognition, fast behavioral reactions against intruders appear to be ensured by exploiting the coarse discrimination level achieved early during odor processing, which is of crucial importance in colony defense. Such an emphasis on speed could result in identification mistakes, i.e. nestmates could potentially be considered as intruders and attacked. However, in ants such as *Camponotus sp.*, attacks start with threats (mandible opening), bites and immobilization and do not result in immediate death – workers guarding narrow nest entrances should therefore have more time to confirm or infirm their original reaction and release nestmates in case of a false alarm. On the other hand, more sophisticated levels of recognition observed in other social insect species, such as within-colony recognition of caste [55], social and/or fertility status [56]–[58] and individual recognition [59], do not require fast reactions and would benefit from detection of more fine scaled variation in CHC-profiles. This could be achieved through further processing in the antennal lobes [48] and in the higher-order integration centers [6]. For example, in the ant *Pachycondyla inversa*, where within-colony discrimination occurs in the form of worker policing by egg eating, it was shown that a worker needs an average of 8 minutes to make the decision to start killing a worker-laid egg or not [60]. We therefore hypothesize that more complex levels of recognition involve additional information processing steps, in ants in general but also in *C. aethiops* should this species show within-colony recognition abilities, enabling slower but more detailed treatment of recognition cues. Social insects would thus rely on a sophisticated and adaptive dual decision-making system enabling them to emphasize either speed or accuracy as required, just as mammals do.

## Materials and Methods

### Study organism

Six colonies of *Camponotus aethiops* were collected in spring 2007 in Italy (Apennines near Bologna), brought to Copenhagen, Denmark and housed in plastic boxes (27×18×8 cm) with a plaster floor. Water was provided *ad libitum* and ants were fed with diluted honey and mealworms (larvae of *Tenebrio molitor* beetles) three times a week. Colonies were kept under standardized laboratory conditions (24°C; L∶D = 12∶12).

### Response times for nestmate recognition

In order to measure the speed of the reaction of ants upon presentation of a chemical stimulus represented by the CHC-profile of non-nestmates, we cooled individual ants on ice and restrained them in a holder only allowing them to move their antennae and mouth parts. The ant holder consisted in an inverted 0.2ml Eppendorf standard microtest tube, whose apex was cut off. The ant's head was passed through the apical hole of the tube and then fixed with adhesive tape stuck behind the ant's neck (*collum*) pushing the head to the wall of the tube, leaving the mouthparts on the exterior side of the tube wall (for details see [61]). The ants were left undisturbed in a quiet place for one hour in order to let them recover from the anesthesia and habituate to the harness. After resting, the individuals that could actively move their antennae and mandibles (more than 90% of the harnessed individuals) were used for the tests (n = 20).

Each ant was tested either with the CHC-extract of nestmates or with the CHC-extract of non-nestmates (prepared in pentane, as explained below) and with the solvent only (control). The testing solutions were applied on pieces of filter paper introduced into Pasteur pipettes heated to approximately 50°C to increase the volatility of the CHC-extract. The stimulus was applied by blowing a pulse of carbon-filtered humidified air (250 ml/min, pulse duration 0.1 sec) generated by a mechanical stimulus air controller (Syntech Company) through the Pasteur pipette over the ant head from a distance of 1 cm. The stimulus controller was equipped with a red LED that is switched off when the stimulus is blown. The stimulus sequence was: pentane, CHC-extract, pentane again.

The tests were video-taped with a digital video camera (SONY, DCR-SR70E) that records at 25 frames per second, thus each frame corresponded to a time-interval of 40 milliseconds. Videos were watched with the software Adobe Premiere Pro 2.0 allowing single frame analysis. In this way, the latency between the presentation of the stimulus (red-light off) and the aggressive reaction of the ant (mandible opening) could be measured as number of frames.

None of the tested ant reacted aggressively upon presentation of the CHC-extract of nestmates or the solvent, but the ants opened their mandibles upon presentation of the CHC-extract of non-nestmates (within 3 to 4 frames; 120–160 milliseconds).

### Extraction of cuticular hydrocarbons (CHCs) and coating

CHCs were extracted by immersing groups of 10 ant workers, previously killed by freezing, in 1 ml pentane for 10 minutes. Pentane was then allowed to evaporate and extracts were stored at −18°C until used. Extracts were then re-diluted in 100 µl pentane and used to coat microscope slides (experiment 1) or the inside of glass capillaries (1.4 mm diameter, experiments 2 and 3). Solvent was allowed to evaporate, so that the non-volatile cuticular hydrocarbons remained on supports (microscope slides or inner capillary walls). Each capillary was coated with CHCs in a quantity equivalent to half a worker, while each microscope slide was coated with the CHC-extract equivalent to 2.5 workers.

After use in bioassays, a random sample of microscope slides (n = 20) were washed with 100 µl of pentane and extracts were analyzed with an Agilent Technologies 6890N gas chromatograph (capillary column Rtx-5, 30 m×0.25 mm×0.50 µm; Restek, Bellefonte, PA, USA; injector *split-splitless*, carrying gas helium at 1 ml min^−1^); temperature program: from 70°C to 200°C at 30°C min^−1^, and from 200°C to 300°C at 3°C min^−1^. Compounds were identified on the basis of their mass spectra, produced by an Agilent Technologies 5975 inert mass selective detector (70eV electron impact ionization) coupled with the gas chromatograph (GC-MS). Chemical analysis revealed that the microscope slides had been successfully coated with CHCs; no other compounds were detected by GC-MS.

### Exposure of ants to CHC-extracts

In experiment 1 (inside-nest exposure), we housed groups of 20 workers (sub-colonies, n = 24 in total) in plastic boxes (56×77×48 mm) with a plaster floor. In each, we introduced four CHC-coated microscope slides. Ants were allowed to freely investigate the slides during 24 hours before being tested for their discrimination abilities. In experiment 2 (bilateral antennal exposure), individual ant workers were restrained in a device which prevented them from moving their head (Figure 1B) and their antennae were inserted into two glass capillaries coated with colony-specific CHCs during 18 hours. Afterwards, ants were gently released from the restraining device and tested for discrimination abilities. Both experiments were replicated three times using different combinations of colonies (n = 428 and 129 treated workers for experiments 1 and 2 respectively).

In experiment 3 (unilateral antennal exposure), workers' antennae were inserted into two capillaries, similarly to experiment 2, but one capillary was coated with colony-specific CHCs and the other with solvent only (sham treatment). Ants were exposed to the treatment during 18 hours, after which we excised one antenna with fine scissors. The side exposed to CHCs (left or right) and the antenna excised (CHC-exposed or sham-exposed) were pseudo-randomly assigned between subjects. Ants were then gently released from the restraining device and immediately tested for their discrimination abilities in aggression tests. The experiment was replicated three times using different combinations of colonies (n = 192 workers tested in total).

### Aggression tests

Discrimination abilities of treated ants were assessed using standard aggression tests, each worker being tested only once. Aggression tests were carried out in a clean circular arena (Ø 52 mm) with a filter paper floor which was changed after each encounter to avoid chemical marking. At the beginning of each test, one target worker previously killed by freezing was placed inside an open cylinder at the center of the test arena. One treated worker was then released from the exposure design and directly introduced in the test arena, outside of the inner cylinder to prevent any initial contacts with the target. We allowed the treated worker to acclimatize to the design for up to three minutes before we removed the inner cylinder. The behavior of the treated worker towards the target was then recorded for 3 (experiments 1 and 2) or 5 (experiment 3) minutes using the software Etholog 2.2 [62]. We quantified the duration of each of the following actions ranked from minimum to maximum aggression level (*a*): antennal contact and grooming (*a* = 0), mandible opening (*a* = 1), biting (*a* = 2) and gaster flexion (*a* = 3). For each aggression test, an overall aggression index (*AI*) was computed according to the formula [63]:
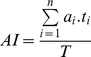
where *a_i_* and *t_i_* are respectively the aggression level and total duration of each action, and *T* is the total interaction time. All experiments were conducted under a blind protocol, i.e. the person who recorded behavior knew neither the treatment experienced by treated workers nor the identity of targets.

### Statistical analyses

After log-transformation, aggression indices were analyzed with a mixed effects linear model (GLMM) using SAS 9.1 (SAS Institute Inc., USA). Whenever main factors or their interaction had a significant effect, i.e. when their associated *P*-value was <0.05, we performed post-hoc comparisons by the method of least square means (see main text for detailed results). The condition of normality of residuals was met for all experiments (Shapiro-Wilk test; experiment 1, *P* = 0.0812; experiment 2, *P* = 0.606; experiment 3, *P* = 0.478).

The model for experiment 1 and 2 included the fixed factors “exposure” (nestmate/non-nestmate odor), “target worker” (nestmate/non-nestmate from stimulus colony/non-nestmate from unrelated alien colony), and the random factor “replicate” (1, 2 or 3) to take into account the possible variation across colonies. We found significant effects of both fixed factors and their interaction (experiment 1: n = 290; exposure, F_1,222_ = 15.66, *P* = 0.0001; target worker, F_2,222_ = 74.41, *P*<0.0001; exposure×target worker, F_2,222_ = 8.79, *P* = 0.0002; experiment 2: n = 129; exposure, F_1,122_ = 10.00, *P* = 0.002; target worker, F_2,122_ = 60.64, *P*<0.0001;exposure×target worker, F_2,122_ = 7.55, *P* = 0.0008).

For experiment 3, the model included the fixed factors “exposure” (nestmate/non-nestmate odor), “remaining antenna” (exposed/non-exposed), “target worker” (nestmate/non-nestmate), and the random factor “replicate” (1, 2 or 3). We found significant effects of the three fixed factors and their interaction (n = 192; exposure, F_1,182_ = 8.86, *P* = 0.0033; remaining antenna, F_1,182_ = 8.88, *P* = 0.0033; target worker, F_1,182_ = 269.11, *P*<0.0001; exposure×remaining antenna×target worker, F_4,182_ = 3.33, *P* = 0.0117).
